# Role of the ubiquitin system and tumor viruses in AIDS-related cancer

**DOI:** 10.1186/1471-2091-8-S1-S8

**Published:** 2007-11-22

**Authors:** Julia Shackelford, Joseph S Pagano

**Affiliations:** 1 Lineberger Comprehensive Cancer Center, The University of North Carolina at Chapel Hill, Chapel Hill, NC, 27599-7295, USA

## Abstract

Tumor viruses are linked to approximately 20% of human malignancies worldwide. This review focuses on examples of human oncogenic viruses that manipulate the ubiquitin system in a subset of viral malignancies; those associated with AIDS. The viruses include Kaposi's sarcoma herpesvirus, Epstein-Barr virus and human papilloma virus, which are causally linked to Kaposi's sarcoma, certain B-cell lymphomas and cervical cancer, respectively. We discuss the molecular mechanisms by which these viruses subvert the ubiquitin system and potential viral targets for anti-cancer therapy from the perspective of this system.

**Publication history: **Republished from Current BioData's Targeted Proteins database (TPdb; ).

## Introduction

Viruses are etiologically linked to approximately 20% of all human malignancies worldwide and much of what we know today about the molecular mechanisms of oncogenesis has come from the study of tumor viruses. The means by which viruses subvert the ubiquitin proteasome system (UPS) is a relatively new area of inquiry. The study of the interactions between viruses and this system not only furthers knowledge of how viruses work, but also often offers shortcuts to understanding cellular processes in general. Though the infectious nature of viruses distinguishes them from other oncogenic factors, it is the adaptation of tumor viruses, mainly DNA viruses, over millennia of co-evolution with their hosts to persistence within these hosts that make the viruses an ideal focus for study of cellular mechanisms reviewed briefly here. This perspective is valid because the immense array of normal intracellular regulatory mechanisms is for the most part intact in latently infected cells, even when they become neoplastic. This symbiosis between virus and cell is mirrored by the fact that in most infected individuals tumors do not develop, and in most instances many years pass between initial infection and appearance of a tumor. The host immune system generally keeps viral infection under control; however, conditions such as acquired immune deficiency syndrome (AIDS) elevate the risk of virus-associated malignancies dramatically [1-4].

Although human immunodeficiency virus (HIV), the cause of AIDS, itself does not have oncogenic properties, the profound immunodeficiency it causes creates a favorable environment for the development of cancer. All HIV-infected patients are at increased risk of developing several types of cancer, particularly in the later stages of AIDS. Despite highly active anti-retroviral therapy (HAART) being widely employed in developed countries, malignancy in this population is still a leading cause of morbidity and mortality [2,5,6].

Among the heterogeneous types of cancer associated with AIDS are Kaposi's sarcoma, immunoblastic B-cell lymphomas and an increased incidence of cervical and anal carcinoma [7,8]. Three human oncogenic viruses are involved causally: Kaposi's sarcoma herpesvirus (KSHV), Epstein-Barr virus (EBV) and human papilloma virus (HPV) [9,10].

AIDS-related malignancies represent only a small portion of all virus-associated human cancers. The consistency of association between a given virus and a specific malignancy ranges from essentially 100% to as low as 15% depending on the virus, the cancer and other factors [11]. Since the UPS regulates diverse cellular functions, including transcription, stress responses, cell cycle, cellular differentiation, angiogenesis, antigen processing and DNA repair [12], it is inevitably involved in oncogenesis induced by all the human tumor viruses [13,14].

Here we discuss several examples of how three tumor viruses manipulate the UPS in AIDS-associated viral malignancies, as well as provide perspectives on UPS-directed agents that might offer pathways to therapeutic intervention in these diseases.

## Kaposi's sarcoma and KSHV

The search for a transmissible infectious agent as the cause of Kaposi's sarcoma led to the discovery in 1994 of KSHV, so far the newest member of the group of identified human oncogenic viruses [15-17]. Even though the incidence of Kaposi's sarcoma has fallen since the introduction of HAART, it is still the most common cancer associated with AIDS [18-21].

The ability to evade immune responses is crucial for long-term survival of viruses in the host. Oncogenic viruses make use of diverse strategies in achieving survival; one is the down-regulation of major histocompatibility complex (MHC) class I antigen presentation through the UPS [22,23].

KSHV encodes several viral products with oncogenic properties, among them two proteins, K3 and K5 (also known as MIR1 and MIR2), that have ubiquitin ligase activity [24,25]. K3 and K5 recruit E2 enzymes with their N-terminal RING-CH domain [25]. Either direct or indirect interactions between the transmembranes of K3 and K5 and MHC class I molecules ultimately lead to the ubiquitylation of lysine residues present in the MHC class I intracytoplasmic tail [25,26]. Ubiquitylated MHC class I molecules are then endocytosed and degraded by the lysosome [25,27,28]. A recent report indicates that K3, but not K5, can promote down-regulation of MHC class I molecules lacking lysine residues in their intracytoplasmic domains [29]. Another study argues that lysine 63-linked ubiquitylation of MHC class I molecules is necessary for their efficient K3 ubiquitin ligase-mediated endolysosomal degradation [30].

Besides K3 and K5, another KSHV product, the immediate-early transcriptional transactivator RTA, is reported to encode E3 ubiquitin ligase activity [31]. RTA-dependent ubiquitylation of interferon regulatory factor 7 (IRF7), a key inducer of interferon-stimulated genes (ISGs), could target it for ubiquitin-dependent proteasomal degradation [31], thus dampening innate immune responses to the infection.

## B-cell lymphomas and EBV

Several decades of intensive studies on EBV, the first human oncogenic virus discovered, have revealed its association with a variety of malignant diseases [11,32-37], including B-cell lymphomas associated with acquired and innate immunosuppressive conditions [38-42].

The EBV product EBNA1 represents an interesting example of how a virus evades immune system responses. EBNA1 is a nuclear protein that binds to EBV episomes and is required for maintenance of latency by the virus [34]. This viral protein contains repeats of Gly-Ala residues that prevent its proteasomal degradation and, additionally, sequester cleaved viral products in a cytoplasmic compartment, rendering them inaccessible for presentation by MHC class I molecules [43]. Although EBNA1 is not the only viral protein expressed during EBV latency, its resistance to UPS-dependent degradation creates a perfect camouflage to prevent recognition by the immune system [43-45].

Like other tumor viruses, EBV demonstrates its oncogenic potential by redirecting cell signaling pathways. Recent studies reveal ways in which EBV can manipulate different components of the UPS. For example, in B-cells its major oncogenic product, latent membrane protein 1 (LMP1), inhibits Siah-1 ubiquitin ligase to rescue the oncogenic factor β-catenin from proteasomal degradation [46,47]. In contrast, in epithelial cells LMP1 activates the same ubiquitin ligase, the targets of which in this case are prolyl hydroxylases (PHDs) [48]. These enzymes mark hypoxia-inducible factor-1α (HIF1α for degradation by the UPS. The stability of PHD 1 and PHD 3 is regulated by both Siah-1 and Siah-2 ubiquitin ligases [49]. The result of LMP1-dependent Siah up-regulation in epithelial cells is that HIFα levels are increased and become active HIFα-responsive genes [48,50]. These are recent observations and physiological reasons for the distinct functional roles of Siah ubiquitin ligases in the different cell types are unknown.

Another EBV latent membrane protein, 2A (LMP2A), acts as a surrogate B-cell receptor by providing constitutive signaling required for B-cell development and survival [51]. LMP2A signaling appears to be regulated in B-cells by association with members of the HECT domain-containing Nedd4 family of ubiquitin ligases [52,53] and likely utilizes ubiquitin-mediated degradation through the proteasome complex to regulate the strength of its own signal. Such processes could allow LMP2A to modulate B-cell pathways such as differentiation, activation or survival [51].

A further EBV latent antigen, EBNA 3C, targets the tumor suppressor pRb for proteasome-dependent degradation through the well known SCF^Skp2^ ubiquitin ligase in different systems including B-cells [54]. Besides directing ubiquitylation that leads to proteasomal degradation, EBV can also affect the regulatory lysine 63 ubiquitylation of IRF7 (the master regulator of type I IFN responses), which leads to its activation instead of degradation [55].

## Cervical carcinoma and HPV

While Kaposi's sarcoma and B-cell lymphoma are the main viral malignancies associated with AIDS, and their connection with HIV infection are hallmarks of the condition, the association between HIV/AIDS and cervical and anal cancer is less obvious [56,57]. However, in 1993 a revised classification system for HIV infection listed invasive cervical cancer as one of the AIDS-defining malignancies [58], and there is growing evidence that HIV infection is associated with increased prevalence and severity of HPV-containing malignant cervical lesions [9,59,60].

More than 95% of all cervical carcinomas contain at least one copy of one of the HPV genotypes 16 & 18 as well as other types that pose a high risk for the malignancy [61]. The HPV *E6* and *E7* genes are the only viral genes that are retained and expressed in tumor tissue, and their role in HPV-induced carcinogenesis is well established [61-63]. Both proteins cause down-regulation of crucial tumor suppressors; E6 inhibits p53 [64-68] and E7 inactivates the retinoblastoma family proteins (pRb) [69-72]. Both E6 and E7 utilize the UPS to target these proteins for degradation and thus inactivation [73]. These interactions are recognized as classic oncogenic mechanisms; they operate in place of mutation of p53 and pRb.

HPV E6 recruits E6-associated protein (E6-AP), now recognized to be an E3 ligase; this E6-E6-AP complex then binds to p53, resulting in E6-AP-mediated ubiquitylation and proteasomal degradation of p53 [67,74,75]. From the perspective of cancer cell biology, this interaction is of interest because the virus product alters endogenous substrate specificity; normally, p53 is a target for Mdm2 ubiquitin ligase-mediated ubiquitylation and degradation [76,77].

The mechanism of E7-induced proteasomal degradation of pRb is still unclear [73,78]. One possibility is that E7 recruits a cellular ubiquitin ligase that targets pRb for ubiquitylation and subsequent degradation. This model is supported by the finding that co-expression of pRb with the Rb-binding-deficient E7 mutant causes a consistent increase in pRb-induced contact-inhibited cell growth in culture [79]. Another possibility is that E7 could function as an adaptor between pRb and the proteasome, thereby targeting pRb directly to the proteasome without prior ubiquitylation, since it has been reported that E7 interacts with the ATPase subunit of the 19S regulatory complex of the 26S proteasome [80].

## Disease models, knockouts and assays

Animal models that mimic human cancers caused by viruses are obviously important for understanding the tumor biology of AIDS-associated malignancies, as well as for evaluating the effect of potential anti-tumor and antiviral drugs. Although there is currently still no animal model that accurately represents KSHV, EBV or HPV pathogenesis, mouse models have been established that attempt to address specific factors known to contribute to the development of the diseases. For example, murine gammaherpesvirus 68 (γHV-68) is used as a rodent model to help understand the pathogenesis of EBV and KSHV. Several reviews of γHV-68 have documented advances made toward understanding the pathogenesis of AIDS-associated malignancies in the context of these two human viruses [81-83].

Another approach is the transplantation of human tumor tissue to mice with severe combined immunodeficiency disease (SCID), which provides valuable models for viral carcinogenesis and also demonstrates the strict species barrier for infection by human viruses [84-87].

As for the roles of the UPS in virus-related cancers, cultured cell lines are still the primary model used at present to study the relations between viral oncogenes and the components of the UPS.

Crucial proof of the transforming potential of KSHV came from *de novo* infection of cultured bone marrow (microvascular) endothelial cells and human umbilical vein endothelial cells (HUVECs). KSHV infection conferred long-term survival of both cell types and anchorage-independent growth of HUVECs [88]. Continuous KSHV infection and also conditional, productive viral replication in cells cultured from primary effusion lymphoma (PEL) (a rare B-cell non-Hodgkin's lymphoma) [89] provide additional models.

The ability of EBV to immortalize normal human B-lymphocytes *in vitro* and to transform them into lymphoblastoid cell lines (LCLs) generates a cell-culture model of AIDS-associated EBV lymphomas [90]. Virus-containing B-lymphoblastoid cell lines that have been derived from primary tumors are also suitable as *in vitro* model systems [91].

Numerous cell lines infected with HPV serve as model cell culture systems to study different aspects of tumorigenesis, but perhaps the most relevant system for evaluating the transforming potential of the HPV oncoproteins is immortalization of primary human keratinocytes, which are the natural host cells of this virus *in vivo*[92]. HPV-immortalized cells are not tumorigenic in nude mice, although they display altered growth and differentiation.

Due to the oncogenic properties of HPV E6 and E7, these proteins have been the focus of most studies on cervical carcinogenesis [64,93-95]. Although the majority of the studies have been performed using cell culture models, several *in vivo* mouse model systems have been developed for the study of HPV-dependent carcinogenesis [96].

## Disease targets and ligands

Both HPV E6 and E7 dysregulate the UPS so that there is down-regulation of the tumor suppressors p53 and pRb. Since both E6 and E7 are immunogenic, these viral products present potential targets for therapeutic vaccines [97-101].

As the UPS is closely involved in the regulation of numerous signaling pathways in tumor cells, it has in the last several years become an attractive target for anti-cancer therapy. The use of proteasome inhibitors to block the final stage in the UPS, proteolysis in the proteasome, presents the opportunity to manipulate intracellular processes in cancer cells for tangible benefit [102-106]. Yet, the functional activity of the UPS is crucial for normal cell function; blockade of protein degradation by proteasome inhibitors causes accumulation of misfolded or damaged proteins, which in turn leads to cell death [107,108]. At the same time, there is much evidence that some proteasome inhibitors are more cytotoxic to proliferating malignant cells than to normal quiescent cells [109].

The first of this new proteasome-inhibiting class of drugs to be on the market, bortezomib (Velcade, formerly known as PS-341), shows promising results in clinical trials with different types of cancer specifically by inhibiting the oncogenic NF-κB signaling pathway [110,111]. Since UPS-dependent degradation of IκB leads to NF-κB activation (as observed in most known malignancies including those that are AIDS-related [112]), bortezomib could be a candidate for the treatment of the virus-related cancers. In *in vitro* studies, bortezomib has demonstrated activity against a variety of malignancies by inducing apoptosis in cancer cells and increasing sensitivity of tumor cells to radiation or chemotherapy [113]. Since bortezomib is proving to be highly efficient for treatment of multiple myeloma and also shows promise for lymphoid cancers [113,114], it could be useful in the treatment of EBV-induced B-lymphomas, which are the second most common AIDS-related malignancy.

Since latent infection with these three DNA viruses is the basis for tumorigenesis, induction of the viral lytic cycle, leading to death of virus-infected malignant cells, is a potential antiviral strategy [115,116]. Recent study shows that bortezomib induces KSHV lytic gene expression *in vitro* in two latently KSHV-infected lymphoma cell lines [117]. This result suggests that the UPS regulates viral reactivation and that proteasome inhibitors could have similar effects on other latently infected virus-associated malignant cells.

Also, targeting of other steps of the UPS, such as specific ubiquitin ligases or deubiquitylating enzymes, could produce more selective effects since ubiquitylating and deubiquitylating complexes specifically bind to potential substrates. KSHV ubiquitin ligases K3 and K5 could be good examples of such targets.

## Next frontiers

The effect of bortezomib and other proteasome inhibitors in virus-associated malignancies needs to be defined further. Viral products themselves are closely involved in UPS-dependent regulation and therefore the effects of proteasome inhibitors can be unexpected. For instance, it has been shown on one hand that proteasome inhibitors inhibit HIV budding [118] and on the other hand that inhibition of proteasome function can enhance HIV-1 infection [119].

Generally, present knowledge of how UPS modulators affect AIDS/HIV-associated or other virus-related malignancies is very limited and calls for further investigation. Recent information on the relations between tumor viruses and the host cell system is summarized in Table 1.

**Table 1:** Viral products manipulate the ubiquitin system in AIDS-related cancers. Summarized here is recent information on the relations between tumor viruses and host cell systems. The general strategy through which the ubiquitin system is manipulated, the effector proteins and the host target proteins are indicated for KSHV, EBV and HPV.

**Malignancies**	Kaposi's sarcoma (KSHV)	Immunoblastic B-cell lymphomas (EBV)	Cervical cancer (HPV)
**Strategy**	Virus-encoded ubiquitin ligases	Dysregulation of host ubiquitin system	Dysregulation of host ubiquitin system
**Viral Effectors**	KSHV K3, K5 and RTA	EBV LMP1	HPV E6 and E7
**Cellular Targets**	MHC class I, IRF7	β-catenin, PHD/HIFa, IRF7	p53, pRb

Despite the limitations of *in vivo* model systems for virus-related human malignancies, some (for example, human peripheral blood lymphocytes (hu-PBL) engrafted in SCID mice) could facilitate screening and preliminary testing of proteasome inhibitors.

Finally, there is no doubt that in the broader panorama of other cancers associated with viruses, such as human T-cell lymphotropic virus-1 (HLTV-1: leukemia), hepatitis B and C viruses (HBV and HCV: hepatocellular carcinoma), as well as EBV (nasopharyngeal and gastric carcinomas, Burkitt's and Hodgkin's lymphomas) and HPV (cervical cancer) in non-immunocompromised patients, many aspects of the UPS are at work and will offer targets for therapy.

## Competing interests

The authors declare that they have no competing interests.

## Publication history

Republished from Current BioData's Targeted Proteins database (TPdb; ).

## References

[B1] Ambinder R. F. (2003). Viruses as potential targets for therapy in HIV-associated malignancies. Hematol Oncol Clin North Am.

[B2] Cheung M. C., Pantanowitz L., Dezube B. J. (2005). AIDS-related malignancies: emerging challenges in the era of highly active antiretroviral therapy. Oncologist.

[B3] Goedert J. J. (2000). The epidemiology of acquired immunodeficiency syndrome malignancies. Semin Oncol.

[B4] Shah M. H., Porcu P., Mallery S. R., Caligiuri M. A. (2003). AIDS-associated malignancies. Cancer Chemother Biol Response Modif.

[B5] Armstrong W., Calabrese L., Taege A. J. (2005). HIV update 2005: origins, issues, prospects, and complications. Cleve Clin J Med.

[B6] Gates A. E., Kaplan L. D. (2002). AIDS malignancies in the era of highly active antiretroviral therapy. Oncology (Williston Park).

[B7] Bellan C., De Falco G., Lazzi S., Leoncini L. (2003). Pathologic aspects of AIDS malignancies. Oncogene.

[B8] Bower M., Palmieri C., Dhillon T. (2006). AIDS-related malignancies: changing epidemiology and the impact of highly active antiretroviral therapy. Curr Opin Infect Dis.

[B9] Aoki Y., Tosato G. (2004). Neoplastic conditions in the context of HIV-1 infection. Curr HIV Res.

[B10] Hille J. J., Webster-Cyriaque J., Palefski J. M., Raab-Traub N. (2002). Mechanisms of expression of HHV8, EBV and HPV in selected HIV-associated oral lesions. Oral Dis.

[B11] Pagano J. S., Blaser M., Buendia M. A., Damania B., Khalili K., Raab-Traub N., Roizman B. (2004). Infectious agents and cancer: criteria for a causal relation. Semin Cancer Biol.

[B12] Ciechanover A., Orian A., Schwartz A. L. (2000). Ubiquitin-mediated proteolysis: biological regulation via destruction. Bioessays.

[B13] Shackelford J., Pagano J. S. (2004). Tumor viruses and cell signaling pathways: deubiquitination versus ubiquitination. Mol Cell Biol.

[B14] Shackelford J., Pagano JS, R. L. J Mayer (2005). Targeting of host-cell ubiquitin pathways by viruses. The Ubiquitin-Proteasome System.

[B15] Bubman D., Cesarman E. (2003). Pathogenesis of Kaposi's sarcoma. Hematol Oncol Clin North Am.

[B16] Viejo-Borbolla A., Ottinger M., Schulz T. F. (2004). Human herpesvirus 8: biology and role in the pathogenesis of Kaposi's sarcoma and other AIDS-related malignancies. Curr HIV/AIDS Rep.

[B17] Viejo-Borbolla A., Schulz T. F. (2003). Kaposi's sarcoma-associated herpesvirus (KSHV/HHV8): key aspects of epidemiology and pathogenesis. AIDS Rev.

[B18] Aversa S. M., Cattelan A. M., Salvagno L., Crivellari G., Banna G., Trevenzoli M., Chiarion-Sileni V., Monfardini S. (2005). Treatments of AIDS-related Kaposi's sarcoma. Crit Rev Oncol Hematol.

[B19] Dittmer D. P., Vahrson W., Staudt M., Hilscher C., Fakhari F. D. (2005). Kaposi's sarcoma in the era of HAART-an update on mechanisms, diagnostics and treatment. AIDS Rev.

[B20] Stebbing J., Sanitt A., Nelson M., Powles T., Gazzard B., Bower M. (2006). A prognostic index for AIDS-associated Kaposi's sarcoma in the era of highly active antiretroviral therapy. Lancet.

[B21] Vanni T., Sprinz E., Machado M. W., Santana R. D., Fonseca B. A., Schwartsmann G. (2006). Systemic treatment of AIDS-related Kaposi sarcoma: Current status and perspectives. Cancer Treat Rev.

[B22] Reinstein E. (2004). Immunologic aspects of protein degradation by the ubiquitin-proteasome system. Isr Med Assoc J.

[B23] Rivett A. J., Hearn A. R. (2004). Proteasome function in antigen presentation: immunoproteasome complexes, Peptide production, and interactions with viral proteins. Curr Protein Pept Sci.

[B24] Benichou S., Benmerah A. (2003). The HIV nef and the Kaposi-sarcoma-associated virus K3/K5 proteins: “parasites” of the endocytosis pathway]. Med Sci (Paris).

[B25] Coscoy L., Sanchez D. J., Ganem D. (2001). A novel class of herpesvirus-encoded membrane-bound E3 ubiquitin ligases regulates endocytosis of proteins involved in immune recognition. J Cell Biol.

[B26] Sanchez D. J., Coscoy L., Ganem D. (2002). Functional organization of MIR2, a novel viral regulator of selective endocytosis. J Biol Chem.

[B27] Coscoy L., Ganem D. (2000). Kaposi's sarcoma-associated herpesvirus encodes two proteins that block cell surface display of MHC class I chains by enhancing their endocytosis. Proc Natl Acad Sci U S A.

[B28] Hewitt E. W., Duncan L., Mufti D., Baker J., Stevenson P. G., Lehner P. J. (2002). Ubiquitylation of MHC class I by the K3 viral protein signals internalization and TSG101-dependent degradation. Embo J.

[B29] Cadwell K., Coscoy L. (2005). Ubiquitination on nonlysine residues by a viral E3 ubiquitin ligase. Science.

[B30] Duncan L. M., Piper S., Dodd R. B., Saville M. K., Sanderson C. M., Luzio J. P., Lehner P. J. (2006). Lysine-63-linked ubiquitination is required for endolysosomal degradation of class I molecules. Embo J.

[B31] Yu Y., Wang S. E., Hayward G. S. (2005). The KSHV immediate-early transcription factor RTA encodes ubiquitin E3 ligase activity that targets IRF7 for proteosome-mediated degradation. Immunity.

[B32] Herrmann K., Niedobitek G. (2003). Epstein-Barr virus-associated carcinomas: facts and fiction. J Pathol.

[B33] Raab-Traub N. (2002). Epstein-Barr virus in the pathogenesis of NPC. Semin Cancer Biol.

[B34] Rickinson A., Kieff E., P. M. Howley (2001). Epstein-Barr virus. Virology.

[B35] Tao Q., Young L. S., Woodman C. B., Murray P. G. (2006). Epstein-Barr virus (EBV) and its associated human cancers--genetics, epigenetics, pathobiology and novel therapeutics. Front Biosci.

[B36] Young L. S., Murray P. G. (2003). Epstein-Barr virus and oncogenesis: from latent genes to tumours. Oncogene.

[B37] Young L. S., Rickinson A. B. (2004). Epstein-Barr virus: 40 years on. Nat Rev Cancer.

[B38] Gottschalk S., Rooney C. M., Heslop H. E. (2005). Post-transplant lymphoproliferative disorders. Annu Rev Med.

[B39] Rui L., Goodnow C. C. (2006). Lymphoma and the control of B cell growth and differentiation. Curr Mol Med.

[B40] Shimoyama Y., Nakamura S., Asano N., Oshiro A., Oyama T. (2006). [Epstein-Barr virus (EBV)-associated lymphomas and lymphoproliferative disorders]. Nippon Rinsho.

[B41] Taylor A. L., Marcus R., Bradley J. A. (2005). Post-transplant lymphoproliferative disorders (PTLD) after solid organ transplantation. Crit Rev Oncol Hematol.

[B42] Yin C. C., Medeiros L. J., Abruzzo L. V., Jones D., Farhood A. I., Thomazy V. A. (2005). EBV-associated B- and T-cell posttransplant lymphoproliferative disorders following primary EBV infection in a kidney transplant recipient. Am J Clin Pathol.

[B43] Masucci M. G. (2004). Epstein-Barr virus oncogenesis and the ubiquitin-proteasome system. Oncogene.

[B44] Dantuma N. P., Masucci M. G. (2003). The ubiquitin/proteasome system in Epstein-Barr virus latency and associated malignancies. Semin Cancer Biol.

[B45] Dantuma N. P., Sharipo A., Masucci M. G. (2002). Avoiding proteasomal processing: the case of EBNA1. Curr Top Microbiol Immunol.

[B46] Jang K. L., Shackelford J., Seo S. Y., Pagano J. S. (2005). Up-regulation of beta-catenin by a viral oncogene correlates with inhibition of the seven in absentia homolog 1 in B lymphoma cells. Proc Natl Acad Sci U S A.

[B47] Shackelford J., Maier C., Pagano J. S. (2003). Epstein-Barr virus activates beta-catenin in type III latently infected B lymphocyte lines: association with deubiquitinating enzymes. Proc Natl Acad Sci U S A.

[B48] Kondo S., Seo S. Y., Yoshizaki T., Wakisaka N., Furukawa M., Joab I., Jang K. L., Pagano J. S. (2006). EBV latent membrane protein 1 up-regulates hypoxia-inducible factor 1alpha through Siah1-mediated down-regulation of prolyl hydroxylases 1 and 3 in nasopharyngeal epithelial cells. Cancer Res.

[B49] Nakayama K., Ronai Z. (2004). Siah: new players in the cellular response to hypoxia. Cell Cycle.

[B50] Wakisaka N., Pagano J. S. (2003). Epstein-Barr virus induces invasion and metastasis factors. Anticancer Res.

[B51] Portis T., Ikeda M, Longnecker R (2004). Epstein-Barr virus LMP2A: regulating cellular ubiquitination processes for maintenance of viral latency?. Trends Immunol.

[B52] Ikeda M., Ikeda A., Longan L. C., Longnecker R. (2000). The Epstein-Barr virus latent membrane protein 2A PY motif recruits WW domain-containing ubiquitin-protein ligases. Virology.

[B53] Winberg G., Matskova L., Chen F., Plant P., Rotin D., Gish G., Ingham R., Ernberg I., Pawson T. (2000). Latent membrane protein 2A of Epstein-Barr virus binds WW domain E3 protein-ubiquitin ligases that ubiquitinate B-cell tyrosine kinases. Mol Cell Biol.

[B54] Knight J. S., Sharma N., Robertson E. S. (2005). Epstein-Barr virus latent antigen 3C can mediate the degradation of the retinoblastoma protein through an SCF cellular ubiquitin ligase. Proc Natl Acad Sci U S A.

[B55] Huye L., Ning S., Pagano JS. (2007). Interferon Regulatory Factor 7 is Activated by a Viral Oncoprotein through RIP-Dependent Ubiquitination. Mol Cell Biol.

[B56] Goedert J. J., Cote T. R., Virgo P., Scoppa S. M., Kingma D. W., Gail M. H., Jaffe E. S., Biggar R. J. (1998). Spectrum of AIDS-associated malignant disorders. Lancet.

[B57] Rabkin C. S., Biggar R. J., Baptiste M. S., Abe T., Kohler B. A., Nasca P. C. (1993). Cancer incidence trends in women at high risk of human immunodeficiency virus (HIV) infection. Int J Cancer.

[B58] (1992). 1993 revised classification system for HIV infection and expanded surveillance case definition for AIDS among adolescents and adults. MMWR Recomm Rep.

[B59] Del Mistro A., Chieco Bianchi L. (2001). HPV-related neoplasias in HIV-infected individuals. Eur J Cancer.

[B60] Nicol A. F., Fernandes A. T., Bonecini-Almeida Mda G. (2005). Immune response in cervical dysplasia induced by human papillomavirus: the influence of human immunodeficiency virus-1 co-infection -- review. Mem Inst Oswaldo Cruz.

[B61] zur Hausen H. (2000). Papillomaviruses causing cancer: evasion from host-cell control in early events in carcinogenesis. J Natl Cancer Inst.

[B62] de Villiers E. M., Fauquet C., Broker T. R., Bernard H. U., zur Hausen H. (2004). Classification of papillomaviruses. Virology.

[B63] Palefsky J. (2006). Biology of HPV in HIV infection. Adv Dent Res.

[B64] Huibregtse J. M., Beaudenon S. L. (1996). Mechanism of HPV E6 proteins in cellular transformation. Semin Cancer Biol.

[B65] Mantovani F., Banks L. (1999). The interaction between p53 and papillomaviruses. Semin Cancer Biol.

[B66] Scheffner M., Werness B. A., Huibregtse J. M., Levine A. J., Howley P. M. (1990). The E6 oncoprotein encoded by human papillomavirus types 16 and 18 promotes the degradation of p53. Cell.

[B67] Thomas M., Pim D., Banks L. (1999). The role of the E6-p53 interaction in the molecular pathogenesis of HPV. Oncogene.

[B68] Tommasino M., Accardi R., Caldeira S., Dong W., Malanchi I., Smet A., Zehbe I. (2003). The role of TP53 in Cervical carcinogenesis. Hum Mutat.

[B69] Dyson N., Howley P. M., Munger K., Harlow E. (1989). The human papilloma virus-16 E7 oncoprotein is able to bind to the retinoblastoma gene product. Science.

[B70] Helt A. M., Galloway D. A. (2003). Mechanisms by which DNA tumor virus oncoproteins target the Rb family of pocket proteins. Carcinogenesis.

[B71] Howley P. M., Munger K., Romanczuk H., Scheffner M., Huibregtse J. M. (1991). Cellular targets of the oncoproteins encoded by the cancer associated human papillomaviruses. Princess Takamatsu Symp.

[B72] Munger K., Werness B. A., Dyson N., Phelps W. C., Harlow E., Howley P. M. (1989). Complex formation of human papillomavirus E7 proteins with the retinoblastoma tumor suppressor gene product. Embo J.

[B73] Scheffner M., Whitaker N. J. (2003). Human papillomavirus-induced carcinogenesis and the ubiquitin-proteasome system. Semin Cancer Biol.

[B74] Talis A. L., Huibregtse J. M., Howley P. M. (1998). The role of E6AP in the regulation of p53 protein levels in human papillomavirus (HPV)-positive and HPV-negative cells. J Biol Chem.

[B75] Thomas M., Banks L. (1998). Inhibition of Bak-induced apoptosis by HPV-18 E6. Oncogene.

[B76] Hengstermann A., Linares L. K., Ciechanover A., Whitaker N. J., Scheffner M. (2001). Complete switch from Mdm2 to human papillomavirus E6-mediated degradation of p53 in cervical cancer cells. Proc Natl Acad Sci U S A.

[B77] Honda R., Tanaka H., Yasuda H. (1997). Oncoprotein MDM2 is a ubiquitin ligase E3 for tumor suppressor p53. FEBS Lett.

[B78] Wang J., Sampath A., Raychaudhuri P., Bagchi S. (2001). Both Rb and E7 are regulated by the ubiquitin proteasome pathway in HPV-containing cervical tumor cells. Oncogene.

[B79] Gonzalez S. L., Stremlau M., He X., Basile J. R., Munger K. (2001). Degradation of the retinoblastoma tumor suppressor by the human papillomavirus type 16 E7 oncoprotein is important for functional inactivation and is separable from proteasomal degradation of E7. J Virol.

[B80] Berezutskaya E., Bagchi S. (1997). The human papillomavirus E7 oncoprotein functionally interacts with the S4 subunit of the 26 S proteasome. J Biol Chem.

[B81] Gasper-Smith N., Bost K. L. (2004). Initiation of the host response against murine gammaherpesvirus infection in immunocompetent mice. Viral Immunol.

[B82] Mistrikova J., Raslova H., Mrmusova M., Kudelova M. (2000). A murine gammaherpesvirus. Acta Virol.

[B83] Simas J. P., Efstathiou S. (1998). Murine gammaherpesvirus 68: a model for the study of gammaherpesvirus pathogenesis. Trends Microbiol.

[B84] Amado R. G., Mitsuyasu R. T., Zack J. A. (1999). Gene therapy for the treatment of AIDS: animal models and human clinical experience. Front Biosci.

[B85] Bonyhadi M. L., Kaneshima H. (1997). The SCID-hu mouse: an in vivo model for HIV-1 infection in humans. Mol Med Today.

[B86] Mosier D. E. (1996). Modeling AIDS in a mouse. Hosp Pract (Minneap).

[B87] Trimble J. J., Salkowitz J. R., Kestler H. W. (2000). Animal models for AIDS pathogenesis. Adv Pharmacol.

[B88] Flore O., Rafii S., Ely S., O'Leary J. J., Hyjek E. M., Cesarman E. (1998). Transformation of primary human endothelial cells by Kaposi's sarcoma-associated herpesvirus. Nature.

[B89] Dourmishev L. A., Dourmishev A. L., Palmeri D., Schwartz R. A., Lukac D. M. (2003). Molecular genetics of Kaposi's sarcoma-associated herpesvirus (human herpesvirus-8) epidemiology and pathogenesis. Microbiol Mol Biol Rev.

[B90] Nilsson K. (1992). Human B-lymphoid cell lines. Hum Cell.

[B91] Drexler H. G., MacLeod R. A. (2003). Leukemia-lymphoma cell lines as model systems for hematopoietic research. Ann Med.

[B92] Munger K., Howley P. M. (2002). Human papillomavirus immortalization and transformation functions. Virus Res.

[B93] Finzer P., Aguilar-Lemarroy A., Rosl F. (2002). The role of human papillomavirus oncoproteins E6 and E7 in apoptosis. Cancer Lett.

[B94] McGlennen R. C. (2000). Human papillomavirus oncogenesis. Clin Lab Med.

[B95] Zwerschke W., Jansen-Durr P. (2000). Cell transformation by the E7 oncoprotein of human papillomavirus type 16: interactions with nuclear and cytoplasmic target proteins. Adv Cancer Res.

[B96] Eckert R. L., Crish J. F., Balasubramanian S., Rorke E. A. (2000). Transgenic animal models of human papillomavirus-dependent disease (Review). Int J Oncol.

[B97] Christensen N. D. (2005). Emerging human papillomavirus vaccines. Expert Opin Emerg Drugs.

[B98] Govan V. A. (2005). Strategies for human papillomavirus therapeutic vaccines and other therapies based on the e6 and e7 oncogenes. Ann N Y Acad Sci.

[B99] Kim S. W., Yang J. S. (2006). Human papillomavirus type 16 E5 protein as a therapeutic target. Yonsei Med J.

[B100] Mahdavi A., Monk B. J. (2005). Vaccines against human papillomavirus and cervical cancer: promises and challenges. Oncologist.

[B101] Shillitoe E. J. (2006). Papillomaviruses as targets for cancer gene therapy. Cancer Gene Ther.

[B102] Elliott P. J., Ross J. S. (2001). The proteasome: a new target for novel drug therapies. Am J Clin Pathol.

[B103] Kisselev A. F., Goldberg A. L. (2001). Proteasome inhibitors: from research tools to drug candidates. Chem Biol.

[B104] Monneret C., Buisson J. P., Magdelenat H. (2005). [A new therapy with bortezomib, an oncologic medicinal product of the year 2004]. Ann Pharm Fr.

[B105] Rajkumar S. V., Richardson P. G., Hideshima T., Anderson K. C. (2005). Proteasome inhibition as a novel therapeutic target in human cancer. J Clin Oncol.

[B106] Tsukamoto S., Yokosawa H. (2006). Natural products inhibiting the ubiquitin-proteasome proteolytic pathway, a target for drug development. Curr Med Chem.

[B107] Adams J. (2004). The proteasome: a suitable antineoplastic target. Nat Rev Cancer.

[B108] Goldberg A. L., Rock K. (2002). Not just research tools--proteasome inhibitors offer therapeutic promise. Nat Med.

[B109] Chauhan D., Hideshima T., Anderson K. C. (2005). Proteasome inhibition in multiple myeloma: therapeutic implication. Annu Rev Pharmacol Toxicol.

[B110] Richardson P. G., Mitsiades C., Hideshima T., Anderson K. C. (2005). Proteasome inhibition in the treatment of cancer. Cell Cycle.

[B111] Zavrski I., Jakob C., Schmid P., Krebbel H., Kaiser M., Fleissner C., Rosche M., Possinger K., Sezer O. (2005). Proteasome: an emerging target for cancer therapy. Anticancer Drugs.

[B112] Pande V., Ramos M. J. (2005). NF-kappaB in human disease: current inhibitors and prospects for de novo structure based design of inhibitors. Curr Med Chem.

[B113] Richardson P. G., Mitsiades C., Hideshima T., Anderson K. C. (2006). Bortezomib: proteasome inhibition as an effective anticancer therapy. Annu Rev Med.

[B114] Orlowski R. Z., Zeger E. L. (2006). Targeting the proteasome as a therapeutic strategy against haematological malignancies. Expert Opin Investig Drugs.

[B115] Israel B. F., Kenney S. C. (2003). Virally targeted therapies for EBV-associated malignancies. Oncogene.

[B116] Gershburg E., Pagano J. S. (2005). Epstein-Barr virus infections: prospects for treatment. J Antimicrob Chemother.

[B117] Brown H. J., McBride W. H., Zack J. A., Sun R. (2005). Prostratin and bortezomib are novel inducers of latent Kaposi's sarcoma-associated herpesvirus. Antivir Ther.

[B118] Schubert U., Ott D. E., Chertova E. N., Welker R., Tessmer U., Princiotta M. F., Bennink J. R., Krausslich H. G., Yewdell J. W. (2000). Proteasome inhibition interferes with gag polyprotein processing, release, and maturation of HIV-1 and HIV-2. Proc Natl Acad Sci U S A.

[B119] Wei B. L., Denton P. W., O'Neill E., Luo T., Foster J. L., Garcia J. V. (2005). Inhibition of lysosome and proteasome function enhances human immunodeficiency virus type 1 infection. J Virol.

